# Attitudes of pet owners in coastal Oaxaca, Mexico, towards pet ownership and access to care

**DOI:** 10.3389/fvets.2025.1644080

**Published:** 2025-09-17

**Authors:** Rachael Schulte, Guillermo Arcega Castillo, Melinda J. Wilkins

**Affiliations:** ^1^Center for Animal Health and Food Safety, College of Veterinary Medicine, University of Minnesota, Saint Paul, MN, United States; ^2^Veterinary Population Medicine, College of Veterinary Medicine, University of Minnesota, Saint Paul, MN, United States

**Keywords:** free-roaming dogs, sterilization campaigns, zoonotic diseases, veterinary access to care, veterinary capacity building, public health education, sea turtle conservation, dog sterilization

## Abstract

Free-roaming dogs pose risks to human, animal, and environmental health, through zoonotic disease transmission, contribution to parasite life cycles, and predation on vulnerable species. Sterilization campaigns are a common method to reduce free-roaming dog populations. A questionnaire was developed to assess the attitudes and practices of dog owners in coastal Oaxaca, Mexico, regarding pet ownership and access to veterinary care. The primary reasons for owning dogs were companionship and protection, and the most common barriers to care reported were cost and access. The professionalism, knowledge, and communication of veterinarians was rated highly, suggesting veterinarians are a trusted source of information and should play an important role in education efforts around zoonotic diseases and animal care, including population management. Short-term sterilization campaigns are effective at reducing free-roaming dog populations and have demonstrated benefits to local wildlife species through reducing predation. These campaigns, however, do not create sustainable change on their own; building local veterinary surgical capacity is an important need in rural Oaxaca.

## 1 Introduction

A survey conducted in 2021 by the National Statistics Institute of Mexico (INEGI) estimated that the population of pet dogs in Mexico was 42,625,010, and in Oaxaca the population of pet dogs was estimated to be 1,394,967 (1). Estimates of the stray dog population were not available, but it is estimated that only 30% of the dog population in Mexico are owned (1). Free-roaming owned and unowned dogs are common in Mexico, but lack of access to affordable veterinary care presents risks to the health of dogs and humans (2–6). Risks include transmission of parasites and zoonotic diseases, dog aggression towards humans, and ecosystem effects such as predation on sensitive wildlife species like sea turtles (2–5, 7–12). Low rates of sterilization due to cost and cultural beliefs contribute to free-roaming dog populations, with owners of bitches who have litters frequently giving the puppies away rather than increasing the number of dogs in their own household (3–5, 7, 13). The Mexican government administers an annual rabies vaccination campaign, which has resulted in high rates of rabies vaccination among dogs and a marked decrease in rabies cases in the country; however, even vaccinated free-roaming dogs can cause severe trauma and infections through bites (3–7, 9, 13–15).

In 2001, the Palmarito Sea Turtle Rescue in Mazunte, Mexico, established the Mazunte Project, an annual sterilization campaign that provides ovariohysterectomy and gonadectomy services to coastal Oaxacan communities at no charge for dogs and cats (3, 11). Anecdotal evidence from local residents and returning veterinary volunteers of the project report fewer dogs seen on the beaches than in previous years, suggesting that the project’s goals of protecting sea turtles through reduction in stray dog populations are being realized (email communications with Pierre DePorre, DVM, Palmarito Sea Turtle Rescue University Liaison, 2/2/2025 and Rich Rodgers, DVM, annual volunteer surgeon, 5/1/2025, conversation with J Adán Ruiz, RVT, Founder and Executive Director of Biological Reserve Cerro Hermoso, 5/7/2025, and WhatsApp with Adriana Cortés-Gómez, DVM, PhD, Director of Latin America Programs, SEE Turtles, 5/2/2025). The long-term goals of the campaign include creating more sustainable access to affordable veterinary care in coastal Oaxaca, by providing training to Mexican veterinary students and new graduates, establishing a permanent mobile clinic, and establishing a teaching hospital in the area (11), (conversation with J Adán Ruiz, RVT, Founder and Executive Director of Biological Reserve Cerro Hermoso, 5/7/2025).

Shifts in reasons for dog ownership have been previously reported, from predominantly guardian or working dogs to companions (3, 6, 7, 13); as attitudes shift, the demand for veterinary care is also likely to shift, and patterns of dog ownership are likely to change. The objective of this survey was to establish a baseline for owner perceptions of access to and necessity of veterinary care in coastal Oaxaca, as well as to investigate owner attitudes towards dog ownership.

## 2 Methods

### 2.1 Questionnaire development and administration

A questionnaire was developed using Qualtrics software (Provo, UT) (see Supplementary Data Sheet 1). The questionnaire included 44 questions in 8 sections: demographic information (8 questions); care of unowned animals (1 question); questions pertaining to the animal brought to the campaign (3 questions); attitudes and practices around animal ownership (8 questions), vaccination (4 questions), sterilization (5 questions), and parasite prevention (6 questions); and access to care (9 questions). The questionnaire employed a mix of structured and semi-structured questions; the majority of questions were multiple choice or multiple select with text entry boxes for respondents to provide additional detail (see Supplementary Data Sheet 1). The University of Minnesota Institutional Review Board (IRB) determined the survey qualified for IRB exemption.

**Figure 1:**
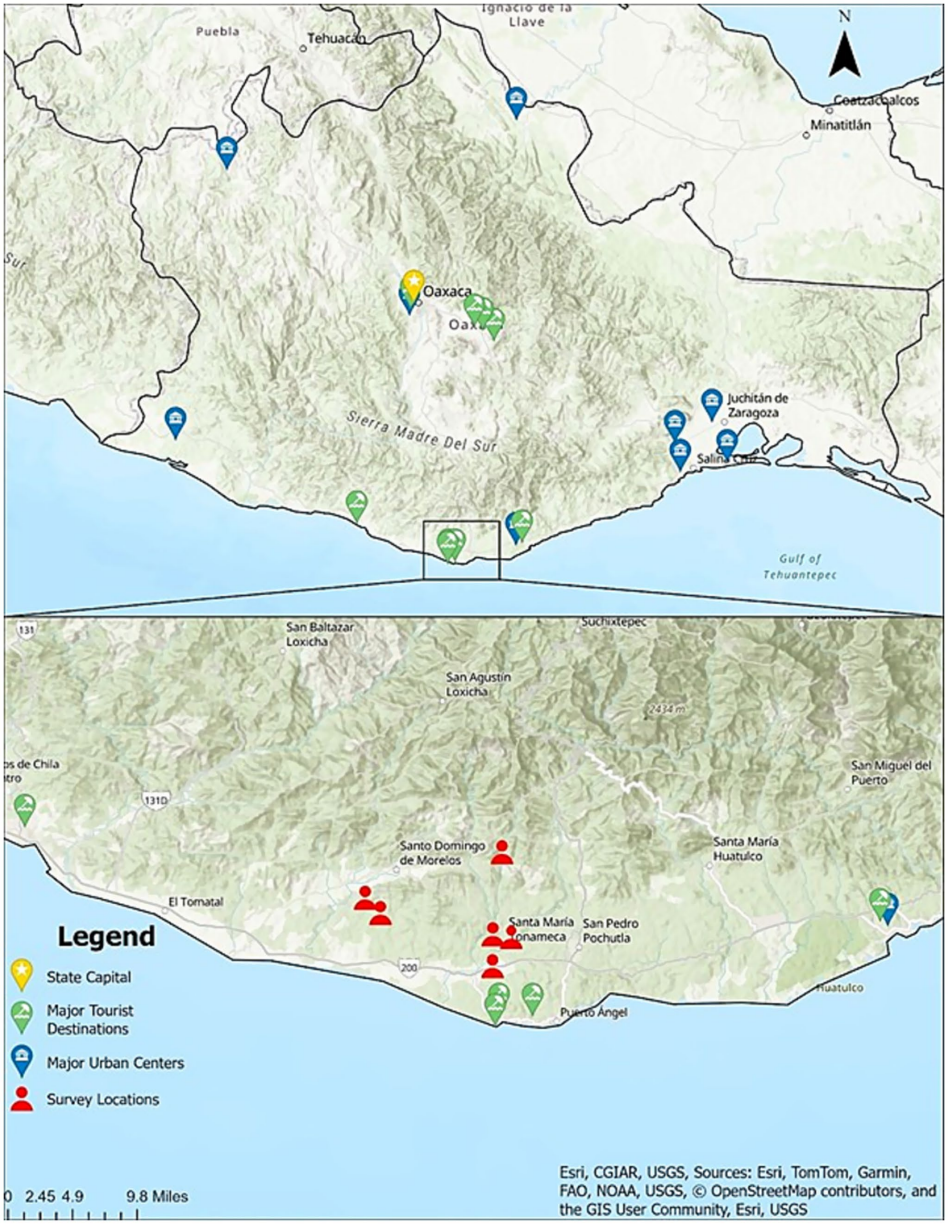
Map of coastal Oaxaca showing the state capital, major tourist destinations and major urban centers. The inset shows survey locations in rural Oaxaca.

The purpose of this questionnaire was to understand attitudes and practices around pet ownership and provide a baseline for local veterinarians and public health officials to use when planning public health interventions. Questionnaires were developed in English, then translated into Spanish by a member of the research team and verified by a veterinarian local to coastal Oaxaca. The questionnaire was administered by interviewers on paper and tablets in Spanish at locations where free sterilization clinics were being offered in coastal Oaxaca in the first 2 weeks of January, 2025 (see Figure 1). Spanish-speaking volunteers were recruited to act as interviewers. Interviewers were trained on-site on the objectives and methodology of the survey and on survey-taking practices. The average duration of interviews was 30 min. Response rate was not recorded.

No questions were required to be answered to complete the survey; missing answers to questions were excluded from analysis and reported percentages are valid percents (missing values were excluded from the calculation). One question regarding income was removed from the survey after the first day of survey administration.

### 2.2 Participants

A convenience sampling strategy was utilized, with the survey being administered to persons taking advantage of free sterilization clinics. Ninety-five surveys were administered. The exclusion criterion was failing to answer any questions related to animal ownership or veterinary care (only answering demographic questions); one survey was excluded, resulting in a final sample size of 94 respondents.

### 2.3 Statistical analysis

Survey analysis was performed initially on Qualtrics (Provo, UT, USA RRID: SCR_016728) and Excel (Redmond, WA, USA RRID: SCR_016137), and finalized in SPSS (Armonk, NY, USA RRID: SCR_016479). Descriptive statistics were used to analyze the results, and the relationships between selected variables were analyzed using chi-square tests due to the small sample size.

## 3 Results

Out of 94 respondents, four were under 18; they took the survey on behalf of related adults, whose ages were not recorded. Of the other 90 responses, 15.6% were 18–24, 15.6% were 25–34, 31.1% were 35–44, 15.6% were 45–54, 8.9% were 55–64, and 13.3% were over 65 years old. As expected, the majority of respondents lived in a rural environment (81.3%, 74/91), with the rest living in an urban environment (18.7%, 17/91). A similar percentage owned their own home (84%, 79/94), while 9.6% (9/94) rented, and 6.4% (6/94) selected “other”; five of those respondents provided text answers, which all reported living with an older related generation, and were 18–32 years old. Less than half of respondents reported someone in their household was under 5 years or over 65 years (40.2%, 37/92). When asked about education level, 25% reported not finishing primary school (23/92), 33.7% reported entering secondary school (31/92), 29.3% completed preparatory or technical education (27/92), 10.9% completed a university degree (10/92), and 1.1% completed post-graduate education (1/92).

The majority of respondents brought an owned animal in to the sterilization campaign, either their own or a relative/neighbor’s animal (91.3%, 84/92), while 4.3% of respondents (4/92) brought in a stray animal, and the remaining 4.3% did not bring an animal to the campaign. If the sterilization campaign was not available, 63% (58/92) stated they would have had their animal sterilized, 19.6% (18/92) stated they might have their animal sterilized, and 17% (16/92) stated they would not have their animal sterilized if the campaign was not offered. When asked about bite history, 97.9% of respondents (92/94) stated none of their dogs or cats had bitten a person hard enough to draw blood out of aggression or fear in the past 2 years, and only 2.1% (2/94) affirmed that their dog or cat had a recent bite history. Mixed breed dogs were owned by the majority of respondents (82.4%, 75/91), 9.6% owned only purebred dogs (9/91), and 7.7% owned both mixed and purebreds (7/91). Dog ownership was reported by 96.8% of respondents (91/94), with a mean of 3.4 and a range of 1 to 9 dogs owned (*n* = 73). Cat ownership was reported by 61.7% of respondents (58/94), with a mean of 2.5 and a range of 1 to 7 cats owned (*n* = 48). Forty-four respondents reported owning chickens (46.8%, *n* = 36). Livestock ownership (cattle, goats, sheep, or pigs) was reported by 13.8% of respondents (13/94, *n* = 9). Four respondents (4.3%) reported ownership of other animals; two owned parrots, and two owned turkeys.

**Figure 2:**
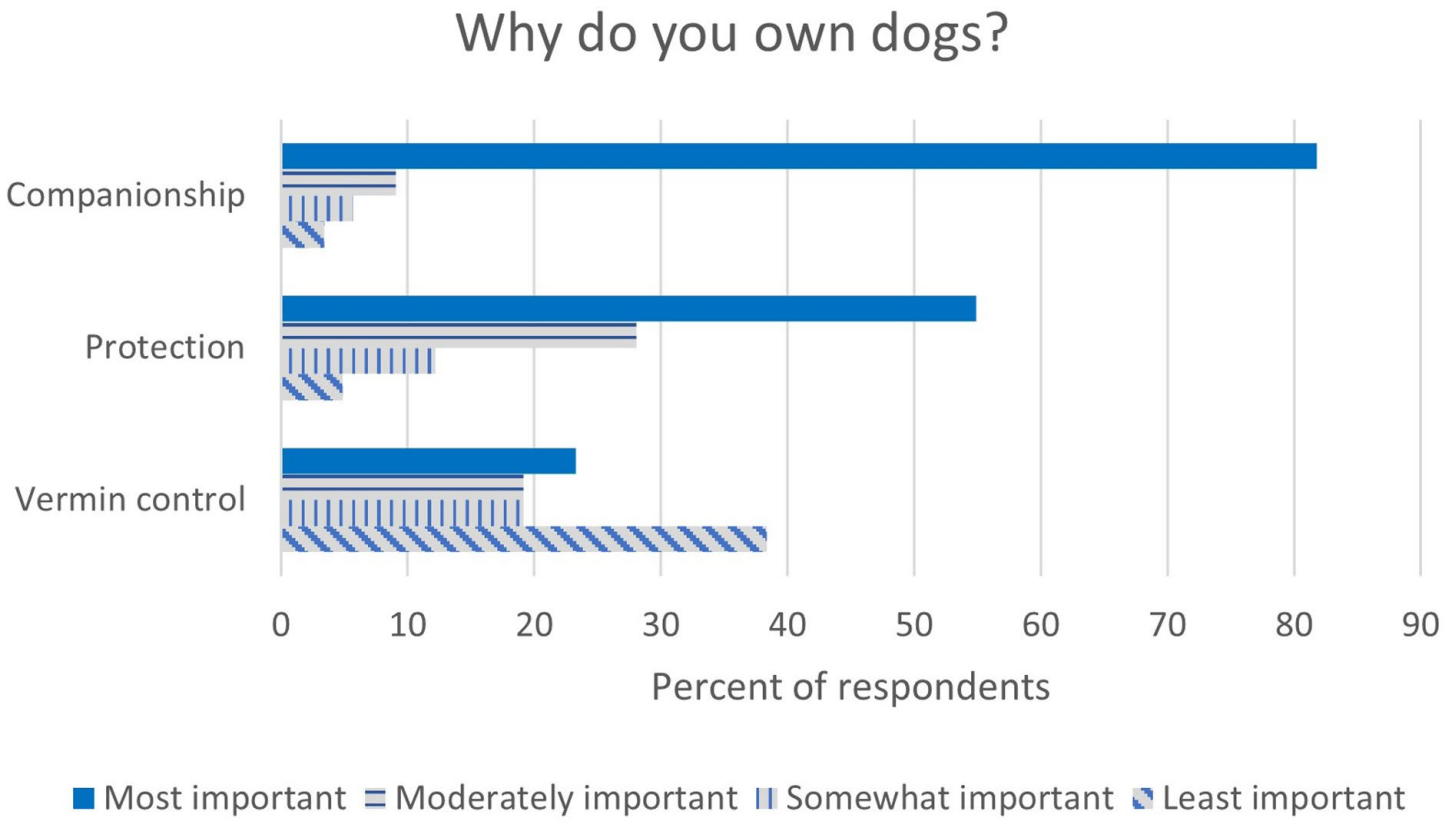
Reasons for owning dogs, ranked from most important to least important, by percentage of respondents.

When asked if they provide care to dogs they do not own, 52.1% (49/94) stated that they do not, while 44.7% (42/94) stated that they feed dogs they do not own. Less than 13% let dogs they do not own into their house or outbuildings, or provide medical care to those dogs. The majority of respondents reported that their dogs and cats were given to them as a gift (54.3%, 51/94); 24.5% (23/94) reported finding their pets, while 23.4% (22/94) reported their pets being born in their house. Less than 20% reported buying or adopting their pets. The majority of respondents (85.1%, 80/94) stated that they always or sometimes allowed their dogs in the house, while 14.9% (14/94) stated that they never let their dogs in the house or only some of their dogs were permitted in the house. Most respondents reported their dogs are in a fenced yard when outside (70.2%, 66/94), while 42.6% (41/94) reported their dogs roam freely for at least part of the day; less than 13% of respondents reported leash walking or tying their dogs when outside. Companionship was rated as the most important reason for owning dogs by 81.8% (72/88) of respondents, followed by protection (54.8%, 45/82) and vermin control (23.3%, 17/73) (see Figure 2). The most common dog-animal interactions were neighbor’s animals (82.3%, 65/79), followed by stray dogs (26.6%, 20/79) and wildlife (19%, 15/79).

**Figure 3:**
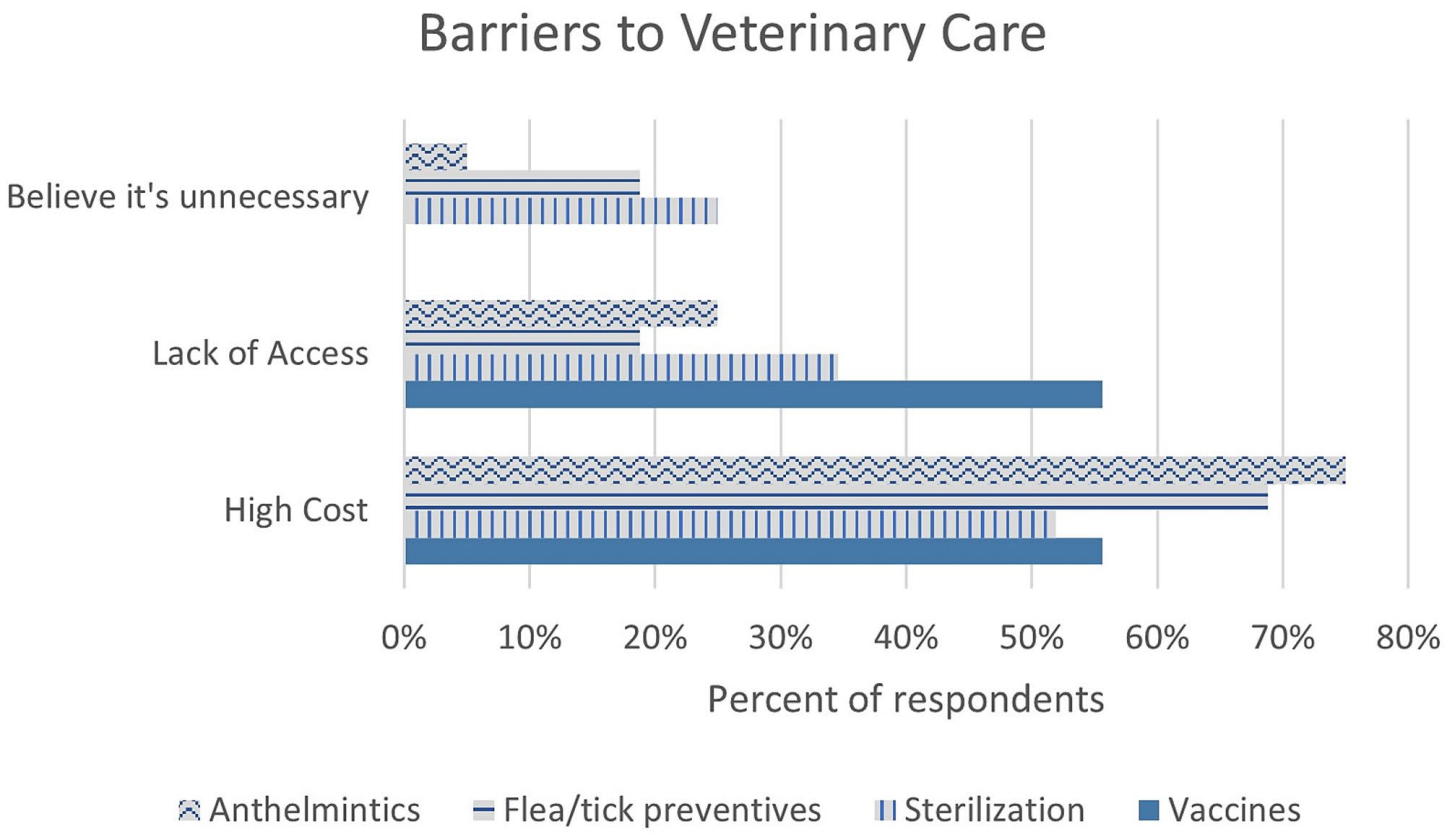
Reasons why specific veterinary services were not obtained for dogs, by percent of respondents. No respondent indicated a belief that vaccines were unnecessary.

Vaccination rates were reported to be high, with 74.4% (67/90) of respondents stating all their dogs were vaccinated, and only 12.2% (11/90) stating none of their dogs were vaccinated. The most common barriers to vaccination were reported to be cost (55.6%, 10/18) and access to services (55.6%, 10/18) (see Figure 3). Rabies was the most common vaccine given (90.4%, 75/83), followed by canine distemper (62.7%, 52/83); *Bordetella* and Leptospirosis were both reported at 9.6% (8/83). The most common provider of vaccination was a government initiative (65.9%, 54/82), followed by private veterinarians (57.3%, 47/82); less than 10% of respondents vaccinated their dogs at home.

Sterilization was less common than vaccination, with 42.4% (39/92) of respondents stating none of their dogs were sterilized, and only 20.7% (19/92) claiming all their dogs were sterilized. Cost (51.9%, 27/52) and access (34.6%, 18/52) were reported to be the most common barriers to sterilization; notably, 25% of respondents did not believe sterilization was necessary (13/52) (see Figure 3). Most respondents did not believe it is necessary to wait to sterilize a bitch until she has had a litter (72.1%, 62/86). The most common provider of sterilization was sterilization campaigns (81.7%, 49/66), followed by private veterinarians (28.3%, 17/66). Sterilization campaigns were considered beneficial to the community by 100% of respondents (91/91).

Flea and tick ectoparasiticides were most commonly given every 3–4 months (44.9%, 40/89) or when external parasites were seen on pets or in the house (24.7%, 22/89). Anthelmintics were given most commonly every 3–4 months (47.7%, 41/86) or seasonally (18.6%, 16/86); the most common barriers to ectoparasiticides and anthelmintics was cost (68.8%, 11/16, and 71.4%, 15/21, respectively) (see Figure 3). The majority of respondents reported picking up their dog’s feces in their yard (88.8%, 79/89), while only 22.5% (20/89) picked up their dog’s feces in the street. Handwashing between handling pets and eating was common, reported by 96.7% of respondents (89/92).

The majority of respondents visited private veterinarians for medical advice, care, or products (53.3%, 49/92), while 26.1% (24/92) visited both a veterinarian and a *granero*^1^. Those who bought products from the granero bought ectoparasiticides (82.5%, 52/63) or anthelmintics (79.4%, 50/63) more commonly than other products or services. The most common services purchased from veterinarians were vaccines (63.6%, 49/77), anthelmintics (57.1%, 44/77), and care for mild illnesses (41.6%, 32/77).

**Figure 4:**
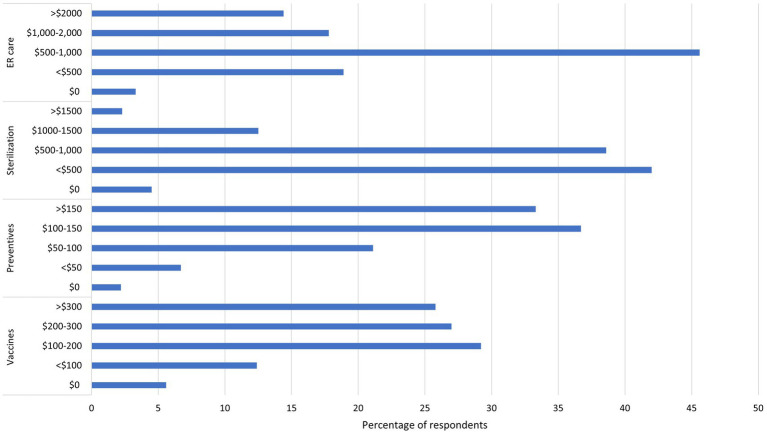
Percent of respondents willing to pay defined amounts for various veterinary services, in Mexican pesos. The amounts listed for vaccines are amount per pet per year, anti-parasitic medications are amount per pet per month, and sterilization is per procedure.

Respondents were asked to rate local veterinarians’ professionalism, knowledge, and communication on a scale of 1–10, with 1 being poor and 10 being excellent. The mean scores were 8.87 for professionalism (*n* = 55); 8.76 for knowledge (*n* = 54); and 9 for communication (*n* = 54). The range for all three was 5–10, and the mode for all three was 10. Respondents were also asked how much they were willing to pay for vaccines, anti-parasitic medications, sterilization, and emergency care for their pets (see Figure 4). The most common veterinary care provided at home by respondents was deworming (77.8%, 70/90), followed by basic wound care (44.4%, 40/90) and vaccinations (30%, 27/90).

Having at least 6 years of education was significantly correlated with having at least some of their dogs vaccinated (Chi-square 5.256, *p* < 0.05, *n* = 88). Living in a rural environment was significantly correlated with owning dogs for protection (Chi-square 16.304, *p* < 0.001, *n* = 80), having at least some of their dogs being vaccinated (Chi-square 6.145, *p* < 0.05, *n* = 87) and sterilized (Chi-square 8.771, *p* < 0.05, n = 89) (see Supplementary Table 1). No significance was found when comparing education or living environment (urban vs. rural) to willingness to seek sterilization outside a campaign, allowing dogs in the house, breed owned, reasons for owning dogs, willingness to seek other veterinary care (vaccines, ectoparasiticides, anthelmintics), seeking care from a veterinarian vs. granero, or willingness to pay more or less for veterinary services. No correlation was found when comparing the presence of children or seniors in the home with bite history, allowing dogs in the house, reasons for owning dogs, or veterinary care metrics.

## 4 Discussion

Although living in close proximity to dogs and cats provides many health benefits (16), it also increases the risk of zoonotic diseases. Immunocompromised persons are at higher risk for complications secondary to zoonotic diseases (17, 18), including children under 5 years and adults over 65 years. Steps to reduce the risk of zoonotic transmission include using anti-parasitic medications appropriately, appropriate disposal of feces, and appropriate hand hygiene. Although high rates of fecal pick up were reported in people’s own yards, the rates dropped off for picking up dog’s feces on the street; this is likely in part due to dogs being allowed to roam freely, and owners not seeing where their dogs defecate. This contributes to the lifecycle of parasites in the environment, especially on beaches, where people often walk barefoot and may become infected (2, 8, 19, 20). The majority of respondents used both ectoparasiticides and anthelmintics every 3–4 months; the similar schedule may be in part due to using combination products. This schedule is unlikely to fully control established flea infestations, but does align with recommendations for anthelmintic administration (21, 22); the schedule may also be in part due to barriers to using anti-parasitic medications, of which cost and access to care were most common. In Puerto Escondido, Oaxaca, Mexico, the most prevalent intestinal parasites were *Toxocara canis*, which was found in 48% of samples (23); in the nearby state Tabasco, Mexico, the prevalence of ticks on dogs was 22% and fleas was 8.7% (24), exemplifying the need for parasite control in this region. Veterinarians and local public health officials should encourage appropriate use of anti-parasitic medications and other steps to prevent zoonotic diseases, including appropriate disposal of feces. The assessment showed high rates of handwashing, the importance of which should continue to be emphasized in conversations about parasites and zoonotic risk. Most respondents reported that they would or might have their pets sterilized even if the campaign was not offered; this suggests the community recognizes the value of sterilization of pets. However, the actual rate of sterilization is lower than the rate of vaccine uptake, and a belief that sterilization was unnecessary for their pets was expressed by some community members. Those who believed sterilization was unnecessary commented that they planned to breed their dogs, or their bitches were spayed so it was unnecessary to sterilize their male dogs; education on reducing unwanted behavior exhibited by intact males, such as urine marking and unplanned mating may be beneficial (2, 5, 25). Such education should take into account a cultural reluctance to sterilize male dogs, and the belief that female dogs should have at least one litter before being sterilized (3, 7, 26).

Interactions with wildlife were reported by numerous respondents, but were likely underreported, as free-roaming dogs would have numerous opportunities to interact with wildlife while unsupervised. Local veterinary and wildlife conservation professionals, and returning volunteers, stated that fewer dogs have been seen on beaches; they stated that more sea turtle hatchlings are successfully reaching the ocean, due to the campaign, education of community members on keeping dogs confined, and efforts to protect nests before hatching (email communications with Pierre DePorre, DVM, Palmarito Sea Turtle Rescue University Liaison, 2/2/2025, and Rich Rodgers, DVM, annual volunteer surgeon, 5/1/2025, conversation with J Adán Ruiz, RVT, Founder and Executive Director of Biological Reserve Cerro Hermoso, 5/7/2025, and WhatsApp with Adriana Cortés-Gómez, DVM PhD, Director of Latin America Programs, SEE Turtles, 5/2/2025). One location that the sterilization campaign has only been offered at more recently has not seen as noticeable an effect on dog populations on beaches, but a noticeable decrease in the number of litters and an increase in the number of community members taking advantage of the campaign has been noted (email communication with Luis Ángel Rojas Cruz, 5/9/2025), suggesting the campaign’s impacts on sea turtle conservation are not always immediate. Alternatively, in 2001, the first year of the campaign, an estimated 70–80 dogs were noted on La Escobilla beach at hatchling emergence; within a year, less than half that number were noted, and now fewer than 5 dogs predate on sea turtles on that beach (email communication with Rich Rodgers, DVM, annual volunteer surgeon, 5/1/2025). This may be due in part to the sterilization campaign decreasing the dog population, shifts in attitudes around pet ownership and free-roaming dogs, or other causes.

The most common provider of sterilization to this community was free campaigns, and 100% of respondents believe these campaigns provide a benefit. However, external aid provides fewer and shorter-term benefits than community driven efforts (27). The sterilization campaign only operates in communities that do not have access to veterinary surgical services, and stopped operating in one community in 2023 to avoid competing with local veterinarians. In 2025, however, that community reached out asking for the campaign to resume visits. This suggests efforts to act as a bridge only until private veterinarians can begin offering services for long-term population management are not fully effective. One ongoing challenge is the limited surgical training in many Mexican veterinary schools. Students often graduate with little hands-on surgical experience, so sterilization campaigns are one of the few opportunities for them to practice, build confidence, and develop surgical competence. Such capacity building efforts are an integral part of the Mazunte project, with local veterinary students and new graduates participating in the campaigns to learn surgical and anesthetic skills (11, 28); additionally, future plans to build a teaching hospital in Oaxaca (11) will provide more sustainable benefits to the community.

High rates of rabies vaccination were reported, likely due to government rabies vaccination campaigns, which report vaccinating 80% of dogs and cats (14); combined with robust surveillance (15), the risk of rabies zoonosis is low. No cases of canine-transmitted human rabies have occurred since 2006, and Mexico was the first country recognized by the WHO as rabies free (29, 30). Wildlife, especially bats, continue to act as reservoirs, and have been implicated in recent human and canine cases (29–32). Additionally, the low bite history reported decreases the risk further, though unwitnessed bites from free-roaming dogs is a risk (4), and all dog bites pose a risk for severe trauma and infection (9). The percentage of respondents that allow their dogs to roam freely was high, which is supported by the large number of dogs roaming freely through the community witnessed by the authors; previous studies have shown that dogs in coastal Oaxaca frequently roam free, and are often fed by other community members in addition to their owners (3), which is supported by nearly half of respondents feeding dogs they do not own. A high percentage of respondents also reported keeping their dogs in a fenced yard, but authors witnessed dogs easily escaping fenced yards while in Oaxaca, suggesting that at least some fenced dogs may contribute to the free-roaming dog population.

Previous work has demonstrated the most common reasons for owning dogs in Oaxaca, Mexico, are protection and companionship (3), which is supported in this work (see Figure 2), where companionship and protection were most frequently rated as the most important reason to own dogs. This likely exemplifies a shift noted in areas with high tourism towards viewing dogs as companions, rather than solely as protectors, as has been previously reported (3, 13). Increased zooeyia, or health benefits associated with owning animals, is expected to accompany these shifts (16).

The most common barriers to care were cost and access to veterinary services (see Figure 3); this is unsurprising, as the campaign targets rural communities without veterinary care, and previous research in the state of Hidalgo, Mexico, also showed low rates of veterinary care and inability to afford sterilization procedures (33) as does broader work on veterinary care accessibility (34, 35). Recent legislation at the federal level mandates publicly funded veterinary clinics to reduce cost and increase access to veterinary care, but veterinarians in Mexico stated a lack of enforcement and funding provisions reduced its efficacy; many veterinarians were unaware of the legislation a year after it took effect (26, 36, 37), demonstrating further work on accessibility is needed. The authors found no publicly funded clinics or hospitals in Oaxaca.

A perceived lack of necessity of certain types of care was also noted, which should be addressed by local public health officials planning public health education campaigns, and local veterinarians educating clients in the course of providing care. Additional reasons for not pursuing vaccination and sterilization for pets was a belief that their dogs were too young; dog ages were not collected in this survey, but previous studies found community members in the city of Mexicali, Baja California believed dogs under 1 year to be too young for vaccination (13), highlighting an area for education by public health officials and veterinarians. Overall, the professionalism, knowledge, and communication skills of local veterinarians was rated highly, which corresponds with high levels of trust in veterinarians in other countries (38, 39), suggesting they are well positioned to lead education efforts related to animal health and zoonotic disease prevention.

The willingness of respondents to pay for veterinary services tended toward a mid-high range of cost for lower-cost items (vaccines, anti-parasitic medications), and toward lower ranges for high-cost items (sterilization, emergency care) (see Figure 4). These results are particularly interesting, as a survey in Hidalgo in 2022 showed nearly half of community members were unwilling to pay more than $100 MXN for sterilization (33), while our data suggests close to 40% of respondents are willing to pay $500–$1,000 MXN for sterilization. Proximity to Mazunte, a small tourist center, may influence perceptions of welfare (3)(see Figure 1), which could contribute to an increased willingness to pay higher prices for sterilization in this study location. The annual sterilization campaign may have also demonstrated the benefits of sterilization, which could contribute to increased willingness to pay for services. This information could be used as a baseline by local veterinarians and veterinary distributors for cost setting.

Correlation was only significant between a few variables, likely due to our small sample size; statistical power was therefore limited. Respondents reporting completing at least 6 years of education were more likely to have some or all of their dogs vaccinated; this may be due to greater awareness of public health practices, or different socioeconomic conditions improving access to veterinary care. Living in a rural environment was correlated with having some or all of their dogs vaccinated and sterilized, and with owning dogs for protection; this is likely due to using dogs in rural settings as guardians for livestock or property. This aligns with research done in 2012, which demonstrated protection was the predominant reason for dog ownership in both rural and urban Oaxaca (3), and may indicate a divergence between rural and urban attitudes toward dog ownership.

Several limitations were present in this assessment. Due to the small sample size, the significance of correlation between variables may have been under or overestimated. Convenience sampling was used, with participants recruited from community members bringing pets to a sterilization campaign for dogs and cats. This resulted in selecting for people who are more likely to own pets, seek veterinary care for pets, and believe that sterilization of pets serves a purpose; this likely resulted in selection bias. Social desirability bias may have also played a role in respondents’ answers to questions on hygiene and animal care practices. This data should not be used to estimate animal ownership in the area, and is not generalizable to the greater public.

This assessment has several notable strengths. Administering questionnaires at sterilization campaign sites provided the opportunity to engage with a wide range of pet owners and community members, providing insight into animal health practices and attitudes toward pet ownership in the region. The questionnaire also captured a wide range of variables related to pet ownership, access to care, preventive practices, and attitudes toward sterilization, enabling a comprehensive descriptive analysis. Finally, this assessment evaluated context-specific factors relevant to rural coastal communities, such as the interaction between free-roaming dogs and local wildlife, including sea turtles, which are often overlooked in broader national studies. This assessment can be used to inform local public health practice and educational strategies.

## 5 Conclusion

The high levels of trust reported in veterinarians position them as essential leaders in both delivering care and educating communities about zoonotic disease risks and the health benefits of sterilization. Although survey respondents believed the free sterilization campaigns provided benefits to the community, such benefits can only be short-term until local veterinary capacity is built-up. Efforts to manage the population with campaigns should continue, but with a greater emphasis on providing opportunities for local veterinarians and veterinary students to develop the surgical skills necessary to provide surgical sterilization services to the community, to improve public health and animal welfare, and to protect vulnerable wildlife species.

## Data Availability

The original contributions presented in the study are publicly available. This data can be found in the Data Repository for the University of Minnesota (DRUM), https://doi.org/10.13020/6HYG-AS52 and in the Supplementary materials.

## Footnotes

^1^A local agricultural supply store that sells animal products such as balanced feed, veterinary medicines, vaccines, and pest control products, sometimes without direct veterinary oversight.
