# Supplementary material for: Attitudes of pet owners in coastal Oaxaca, Mexico, towards pet ownership and access to care
Source: Front Vet Sci. 2025 Sep 17;12:1644080. doi: 10.3389/fvets.2025.1644080 (PMC12487428; doi:10.3389/fvets.2025.1644080)
Supplement: Supplementary file 1 [file Data_Sheet_1.PDF]

University of Minnesota
Driven to Discover®

English

Demographic information

Q1. First name, last initial

Q2. Age

Q3. City

Q4. Do you live in a rural or urban environment?

- ○ I live in an urban environment (in a town with 2,500 people or more)
- ○ I live in a rural environment (in a town of less than 2,500 people)

Q5. Do you rent or own your residence?

- ○ I rent my residence
- ○ I own my own home
- ○ [ ] Other (please describe)

Q6. Is anyone living in your home under the age of 5 years or over the age of 65 years?

- ○ Yes
- ○ No
- ○ I don't know

Q7. How much education have you completed?

- ○ Did not finish primary school (<6 yr)
- ○ Completed primary and secondary school (6-9 yr)
- ○ Completed preparatory school or technical education (12 yr)
- ○ Completed a university degree (e.g. licenciatura)
- ○ Completed postgraduate education (e.g. master's, doctorate)

Q8. What is your monthly income?

- ○ Less than 3,000 MX
- ○ 3,000-5000 MX
- ○ 5,000-7,000 MX
- ○ Over 7,000 MX

Unowned animals

Q9. Do you take care of stray dogs that you do not own?

- ☐ I do not take care of street or free-roaming dogs.
- ☐ I feed them
- ☐ I let them into an outbuilding like a shed
- ☐ I let them in the house
- ☐ I provide basic medical care (vaccinations, s/n, deworming, basic wound care)
- ☐ I provide advanced medical care (sick visits to vet)

Animal presented to Campaign

Q10. For the animal you brought in today, is it:

- ○ Owned (lives in your/your neighbor's place of residence or yard)
- ○ A stray animal
- ○ I did not bring an animal in today
- ○ ______ Other (please describe)

Q11. What is your pet's name?

______

Q12. If this program were not available, would you have

otherwise have your animal spayed or neutered?

- ○ Yes
- ○ Maybe
- ○ No

Animal Ownership

Q13. What type of animals do you own (as in, they live in your residence or on your property)? How many of each type?

- ☐ [ ] Dog(s)
- ☐ [ ] Cat(s)
- ☐ [ ] Chicken(s)
- ☐ [ ] Livestock (cattle, goats, sheep, pigs)
- ☐ [ ] Other (please describe)
- ☐ I don't own any animals

Q14. How did you acquire your dogs and cats?

- ☐ I bought them from a breeder or store
- ☐ I adopted them from a shelter
- ☐ They were given to me as a gift
- ☐ I found them
- ☐ They were born in my house

☐ [ ] Other (please describe)

Q15. **In the past 2 years**, have any of your dogs or cats bitten a person hard enough to draw blood out of aggression or fear?

- ○ Yes
- ○ No
- ○ I don't know

Q16. Are your dogs allowed in your house?

- ○ Always
- ○ Sometimes
- ○ Never
- ○ Only some are allowed in the house

Q17. When outdoors, my dogs are:

- ☐ Always walked on a leash
- ☐ Loose in a fenced yard
- ☐ Tied or chained outdoors
- ☐ In a kennel
- ☐ Allowed to roam freely throughout the day
- ☐ Allowed to roam freely day and night
- ☐ [ ] Other (please describe)

Q18. Are your dogs purebred or mixed breed?

- ○ Purebred
- ○ Mixed breed
- ○ I own both purebred and mixed breed dogs
- ○ I don't know

Q19. Why do you own dogs? Rank in order of most important reason to least important.

	Most important	Moderately important	Somewhat important	Least important
They provide protection	○	○	○	○
They control/chase away vermin	○	○	○	○
They are a companion	○	○	○	○
Other (please describe, if nothing rank as least important) [ ]	○	○	○	○

Q20. Have you seen your dogs interact with animals that you don't own?

- ☐ My dogs interact with my neighbor's animals
- ☐ My dogs interact with stray dogs
- ☐ My dogs interact with feral cats
- ☐ My dogs interact with wildlife (raccoons, rodents, turtles, etc) - please list

[ ]

- [ ] ____ My dogs interact with other animals (please list)
- [ ] I don't know

Vaccination

Q21. Are your dogs vaccinated?

- ○ All my dogs are vaccinated
- ○ Only some of my dogs are vaccinated
- ○ None of my dogs are vaccinated
- ○ I don't know

Q22. If your dogs are not all vaccinated, why?

- [ ] I can't afford to vaccinate all my dogs
- [ ] Some/all of my dogs aren't old enough for vaccination
- [ ] Some/all of my dogs aren't healthy enough to be vaccinated
- [ ] I don't have access to vaccination services
- [ ] I don't think vaccination is necessary for all my dogs
- [ ] ____ Other (please describe)

Q23. What vaccines have your dogs been given? Check all that apply

- [ ] Rabies
- [ ] Distemper/parvo
- [ ] Bordetella (kennel cough)

☐ Leptospirosis

☐ I don't know

Q24. Who vaccinated your dogs?

☐ Government initiative

☐ Private veterinarian

☐ Someone in my family vaccinated them

☐ [ ] Other (please describe)

Sterilization

Q25. Are your dogs spayed or neutered?

○ All my dogs are spayed/neutered

○ Some of my dogs are spayed/neutered

○ None of my dogs are spayed neutered

○ I don't know

Q26. If your dogs are not all spayed or neutered, why?

☐ I can't afford to spay/neuter all my dogs

☐ Some/all of my dogs aren't old enough for spay/neuter

☐ Some/all of my dogs aren't healthy enough

☐ I don't have access to spay/neuter services

☐ I don't think spay/neuter is necessary for all my dogs

☐ [ ] other

Q27. Do you believe it's necessary to wait until a bitch has had a litter to spay her?

- ○ Yes, always
- ○ Sometimes
- ○ No, I don't believe it's necessary
- ○ I don't know

Q28. Who spayed or neutered your dogs (other than the one(s) you brought in today)?

- ☐ A free spay/neuter campaign at a different time
- ☐ A private veterinarian
- ☐ I don't know
- ☐ I don't have any other dogs
- ☐ ________ Other (please describe)

Q29. Do you believe sterilization campaigns are beneficial for the community?

- ○ Yes, I believe they provide benefits
- ○ No, I don't believe they provide any benefits
- ○ I don't know

Parasite Prevention

Q30. Do you give flea and tick preventives to your dogs?

- ○ I give it every month
- ○ I give it every 3-4 months
- ○ [ ] I only give it seasonally (explain what seasons)
- ○ I only give it when I notice fleas or ticks on my dogs or in my house
- ○ I don't give these medications
- ○ [ ] Other (explain)
- ○ I don't know

Q31. If you don't give these medications, why?

- ☐ I can't afford it
- ☐ I don't have access to medications
- ☐ I don't think it's necessary
- ☐ [ ] Other (please describe)

Q32. Do you give internal parasite (worms) prevention or treatment to your dogs?

- ○ I give it every month
- ○ I give it every 3-4 months
- ○ I only give it seasonally (explain what seasons)
- ○ I only give it when I notice worms on my dogs/ feces
- ○ I only give it when my dogs have diarrhea
- ○ I don't give these medications
- ○ [ ] Other (explain)
- ○ I don't know

Q33. If you don't give these medications, why?

- ☐ I can't afford it
- ☐ I don't have access to medications
- ☐ I don't think it's necessary
- ☐ [ ] Other (please describe)

Q34. Do you pick up and dispose of your dog's feces?

- ☐ Yes, when my dog defecates in my yard
- ☐ Yes, when my dog defecates in the park
- ☐ Yes, when my dog defecates in the street
- ☐ Yes, when my dog defecates in front of my shop
- ☐ I pick up all dog feces in front of my shop
- ☐ I never pick up my dog's feces
- ☐ [ ] Other (please explain)

Q35. Do you wash your hands after touching your pets and before eating?

- ○ Yes, always
- ○ Sometimes
- ○ No, never

Access to care

Q36. **In the last 2 years**, have you sought advice, medical care, or purchased products from a private veterinarian, granero, or both?

- ○ Private Veterinarian
- ○ Granero
- ○ Both
- ○ Neither

Q37. **In the last 2 years**, what care or products have you purchased from a granero for **pets you own**? Provide additional detail on what products or for what purpose you purchased them.

- ☐ [ ] Yes, I had vaccinations given at the granare
- ☐ Yes, I purchased vaccines that I administered to my pets [ ]
- ☐ [ ] Yes, for flea/tick preventives
- ☐ [ ] Yes, for deworming medications
- ☐ [ ] Yes, for advice on mild illness (diarrhea, cough, etc)
- ☐ [ ] Other (please describe)

Q38. **In the last 2 years**, what care have you sought from a private veterinarian **for a pet you own**? Provide additional

detail on what products or for what purpose you purchased them.

- [ ] ☐ Yes, for vaccinations
- [ ] ☐ Yes, for spay/neuter
- [ ] ☐ Yes, for deworming/preventives
- [ ] Yes, for mild illness (Outpatient care with medications) ☐
- [ ] ☐ Yes, for trauma (hit-by-car, animal bite)
- [ ] ☐ Yes, for severe illness (Hospitalization)
- [ ] ☐ Other (please describe)

Q39. How would you rate the performance of veterinarians in your community? Please rate with a scale of 1 being poor performance, and 10 being excellent performance.

- [ ] ☐ Professionalism
- [ ] ☐ Knowledge
- [ ] ☐ Communication

Q40. How much are you willing to pay for vaccinations **for**

each pet per year?

- Less than $100 MXN
- $100 - $200 MXN
- $200 - $300 MXN
- More than $300 MXN
- I am not willing or able to pay for vaccinations

Q41. How much are you willing to pay for preventives/deworming medications **for each pet per month**?

- Less than $50 MXN
- $50 - $100 MXN
- $100 - $150 MXN
- More than $150 MXN
- I am not willing or able to pay for deworming medications

Q42. How much are you willing to pay for a spay/neuter procedure?

- Less than $500 MXN
- $500 - $1,000 MXN
- $1,000 - $1,500 MXN
- More than $1,500 MXN
- I am not willing or able to pay for these procedures

Q43. How much are you willing to pay for emergency care **for**

your pet?

- ○ Less than $500 MXN
- ○ $500 - $1,000 MXN
- ○ $1,000 - $2,000 MXN
- ○ More than $2,000 MXN
- ○ I am not willing or able to pay for emergency care

Q44. **In the past 2 years**, what medical care have you yourself provided **to your pet** in your home?

- ☐ Vaccinations
- ☐ Deworming
- ☐ Basic wound care
- ☐ None
- ☐ [ ] Other (please describe)
