# Supplementary material for: Attitudes of pet owners in coastal Oaxaca, Mexico, towards pet ownership and access to care
Source: Front Vet Sci. 2025 Sep 17;12:1644080. doi: 10.3389/fvets.2025.1644080 (PMC12487428; doi:10.3389/fvets.2025.1644080)
Supplement: Supplementary file 3 [file Table_1.DOCX]

Supplementary Material

# Supplementary Data

Supplementary Table 1: Comparing education to attitudes regarding animal ownership and veterinary care.

|  |  | Education | | Χ^2^ | df | p-value |
| --- | --- | --- | --- | --- | --- | --- |
|  |  | Less than 6 years | More than 6 years |  |  |  |
| Would you have sterilized your dogs if the campaign was not offered? (n=90) | Maybe/yes | 16  17.8% | 58  64.4% | 3.386 | 1 | 0.066 |
|  | No | 7  7.8% | 9  10% |  |  |  |
|  |  |  |  |  |  |  |
| Are your dogs vaccinated? (n=88) | Yes/At least some are vaccinated | 17  19.3% | 60  68.2% | 5.256 | 1 | 0.022 |
|  | None are vaccinated | 6  6.8% | 5  5.7% |  |  |  |
|  |  |  |  |  |  |  |
| Are your dogs sterilized? (n=89) | Yes/at least some are sterilized | 8  9.0% | 42  47.2% | 2.743 | 1 | 0.098 |
|  | None are sterilized | 12  13.5% | 27  30.3% |  |  |  |
|  |  |  |  |  |  |  |
| Do you believe it is necessary to allow a bitch to have a litter before spay? (n=84) | Yes/maybe | 8  9.5% | 16  19.0% | 0.887 | 1 | 0.346 |
|  | No | 14  16.7% | 46  54.8% |  |  |  |
|  |  |  |  |  |  |  |
| Do you give flea and tick preventives? (n=87) | Yes | 19  21.8% | 60  69.0% | 0.696 | 1 | 0.404 |
|  | No | 3  3.4% | 5  5.7% |  |  |  |
|  |  |  |  |  |  |  |
| Do you give deworming medications? (n=84) | Yes | 16  19.0% | 56  66.7% | 2.074 | 1 | 0.150 |
|  | No | 5  6.0% | 8.3% |  |  |  |
|  |  |  |  |  |  |  |
| Do you wash your hands after touching your pets and before eating? (n=90) | Yes | 22  24.4% | 74.4% | 0.327 | 1 | 0.567 |
|  | No | 0  0% | 1  1.1% |  |  |  |
|  |  |  |  |  |  |  |
| How much are you willing to pay for vaccines? (n=88) | Less than 200 MXN/Not willing to pay | 14  15.9% | 27  30.7% | 3.425 | 1 | 0.064 |
|  | More than 200 MXN | 8  9.1% | 39  44.3% |  |  |  |
|  |  |  |  |  |  |  |
| How much are you willing to pay for preventives? (n=88) | Less than 100 MXN/Not willing to pay | 7  8.0% | 19  21.6% | 0.073 | 1 | 0.787 |
|  | More than 100 MXN | 15  17.0% | 47  53.4% |  |  |  |
|  |  |  |  |  |  |  |
| How much are you willing to pay for sterilization? (n=86) | Less than 1000 MXN/Not willing to pay | 18  20.9% | 55  64% | 0.15 | 1 | 0.903 |
|  | More than 1000 MXN | 3  3.5% | 10  11.6% |  |  |  |
|  |  |  |  |  |  |  |
| How much are you willing to pay for emergency care? (n=88) | Less than 1000 MXN/Not willing to pay | 17  8.0% | 42  47.7% | 1.389 | 1 | 0.239 |
|  | More than 1000 MXN | 5  5.7% | 24  27.3% |  |  |  |

Supplementary Table 2: Comparing living environment to attitudes regarding animal ownership and veterinary care.

|  |  | Environment | | Χ^2^ | df | p-value |
| --- | --- | --- | --- | --- | --- | --- |
|  |  | Rural | Urban |  |  |  |
| Would you have sterilized your dogs if the campaign was not offered? (n=89) | Maybe/yes | 59  66.3% | 14  15.7% | 0.397 | 1 | 0.529 |
|  | No | 14  15.7% | 2  2.2% |  |  |  |
|  |  |  |  |  |  |  |
| Have any of your pets bitten a person hard enough to draw blood out of aggression or fear? (n=91) | Yes | 1  1.1% | 1  1.1% | 1.320 | 1 | 0.251 |
|  | No | 73  80.2% | 16  17.6% |  |  |  |
|  |  |  |  |  |  |  |
| Are your dogs allowed in your house? (n=91) | Yes, sometimes | 66  72.5% | 14  15.4% | 0.608 | 1 | 0.436 |
|  | Never | 8  8.8% | 3  3.3% |  |  |  |
|  |  |  |  |  |  |  |
| Are your dogs purebred or mixed breed? (n=88) | Purebred/ I own both | 12  13.6% | 3  3.4% | 0.04 | 1 | 0.841 |
|  | Mixed breed | 60  68.2% | 13  14.8% |  |  |  |
|  |  |  |  |  |  |  |
| I own dogs for protection (n=80) | Most/Moderately important | 61  76.3% | 5  6.3% | 16.304 | 1 | <0.001 |
|  | Somewhat/Not important | 7  8.8% | 7  8.8% |  |  |  |
|  |  |  |  |  |  |  |
| I own dogs to control vermin (n=71) | Most/Moderately important | 25  35.2% | 5  7.0% | 0.094 | 1 | 0.759 |
|  | Somewhat/Not important | 33  46.5% | 8  11.3% |  |  |  |
|  |  |  |  |  |  |  |
| I own dogs for companionship (n=86) | Most/Moderately important | 61  70.9% | 17  19.8% | 2.173 | 1 | 0.140 |
|  | Somewhat/Not important | 8  9.3% | 0  0% |  |  |  |
|  |  |  |  |  |  |  |
| Are your dogs vaccinated? (n=87) | Yes/At least some are vaccinated | 65  74.7% | 11  12.6% | 6.145 | 1 | 0.013 |
|  | None are vaccinated | 6  6.9% | 5  5.7% |  |  |  |
|  |  |  |  |  |  |  |
| Are your dogs sterilized? (n=89) | Yes/at least some are sterilized | 35  39.3% | 15  16.9% | 8.771 | 1 | 0.003 |
|  | None are sterilized | 37  41.6% | 2  2.2% |  |  |  |
|  |  |  |  |  |  |  |
| Do you believe it is necessary to allow a bitch to have a litter before spay? (n=84) | Yes/maybe | 22  26.2% | 2  2.4% | 1.680 | 1 | 0.195 |
|  | No | 48  57.1% | 12  14.3% |  |  |  |
|  |  |  |  |  |  |  |
| Do you give flea and tick preventives? (n=86) | Yes | 62  72.1% | 15  17.4% | 0.038 | 1 | 0.845 |
|  | No | 7  8.1% | 2  2.3% |  |  |  |
|  |  |  |  |  |  |  |
| Do you give deworming medications? (n=84) | Yes | 57  67.9% | 14  16.7% | 0.134 | 1 | 0.714 |
|  | No | 11  13.1% | 2  2.4% |  |  |  |
|  |  |  |  |  |  |  |
| Do you wash your hands after touching your pets and before eating? (n=90) | Yes | 72  80% | 17  18.9% | 0.235 | 1 | 0.627 |
|  | No | 1  1.1% | 0  0% |  |  |  |
|  |  |  |  |  |  |  |
| How much are you willing to pay for vaccines? (n=88) | Less than 200 MXN/Not willing to pay | 36  40.9% | 5  5.7% | 1.850 | 1 | 0.174 |
|  | More than 200 MXN | 36  40.9% | 11  12.5% |  |  |  |
|  |  |  |  |  |  |  |
| How much are you willing to pay for preventives? (n=88) | Less than 100 MXN/Not willing to pay | 22  25% | 4  4.5% | 0.366 | 1 | 0.545 |
|  | More than 100 MXN | 49  55.7% | 13  14.8% |  |  |  |
|  |  |  |  |  |  |  |
| How much are you willing to pay for sterilization? (n=87) | Less than 1000 MXN/Not willing to pay | 60  69 % | 14  16.1% | 0.122 | 1 | 0.727 |
|  | More than 1000 MXN | 10  11.5% | 3  3.4% |  |  |  |
|  |  |  |  |  |  |  |
| How much are you willing to pay for emergency care? (n=88) | Less than 1000 MXN/Not willing to pay | 45  51.1% | 14  15.9% | 2.235 | 1 | 0.135 |
|  | More than 1000 MXN | 26  29.5% | 3  3.4% |  |  |  |
|  |  |  |  |  |  |  |
| Where did you seek care? (n=75) | Granero (pet supply store) | 4  5.3% | 0  0% | 2.214 | 2 | 0.331 |
|  | Private Veterinarian | 36  48% | 11  14.7% |  |  |  |
|  | Both | 21  28% | 3  4% |  |  |  |

Supplementary Table 3: Comparing presence of children and elderly persons in the home with attitudes regarding animal ownership and veterinary care. Age is used as a proxy for immunocompromise in this comparison.

|  |  | Children or elderly in home? | | Χ^2^ | df | p-value |
| --- | --- | --- | --- | --- | --- | --- |
|  |  | No one in the home is under 5 years or over 65 years. | Someone in the home is under 5 years or over 65 years. |  |  |  |
| Have any of your pets bitten a person hard enough to draw blood out of aggression or fear? (n=92) | Yes | 2  2.2% | 0  0% | 1.644 | 1 | 0.200 |
|  | No | 49  53.3% | 41  44.6% |  |  |  |
|  |  |  |  |  |  |  |
| Are your dogs allowed in your house? (n=92) | Yes, sometimes | 46  50% | 36  39.1% | 0.134 | 1 | 0.714 |
|  | Never | 5  5.4% | 5  5.4% |  |  |  |
|  |  |  |  |  |  |  |
| I own dogs for protection (n=80) | Most/Moderately important | 36  45% | 31  38.8% | 0.286 | 1 | 0.605 |
|  | Somewhat/Not important | 8  10% | 5  6.3% |  |  |  |
|  |  |  |  |  |  |  |
| I own dogs to control vermin (n=71) | Most/Moderately important | 19  26.8% | 10  14.1% | 1.213 | 1 | 0.271 |
|  | Somewhat/Not important | 22  31% | 20  28.2% |  |  |  |
|  |  |  |  |  |  |  |
| I own dogs for companionship (n=86) | Most/Moderately important | 46  53.5% | 32  37.2% | 1.365 | 1 | 0.243 |
|  | Somewhat/Not important | 3  3.5% | 5  5.8% |  |  |  |
|  |  |  |  |  |  |  |
| Are your dogs vaccinated? (n=88) | Yes/At least some are vaccinated | 42  47.7% | 35  39.8% | 1.287 | 1 | 0.255 |
|  | None are vaccinated | 8  9.1% | 3  3.4% |  |  |  |
|  |  |  |  |  |  |  |
| Are your dogs sterilized? (n=89) | Yes/at least some are sterilized | 29  32.6% | 21  23.6% | 0.154 | 1 | 0.695 |
|  | None are sterilized | 21  23.6% | 18  20.2% |  |  |  |
|  |  |  |  |  |  |  |
| Do you give flea and tick preventives? (n=87) | Yes | 42  48.3% | 36  41.4% |  |  |  |
|  | No | 4  4.6% | 5  5.7% |  |  |  |
|  |  |  |  |  |  |  |
| Do you give deworming medications? (n=84) | Yes | 40  47.6% | 32  38.1% | 0.032 | 1 | 0.858 |
|  | No | 7  8.3% | 5  6.0% |  |  |  |
|  |  |  |  |  |  |  |
| Do you wash your hands after touching your pets and before eating? (n=90) | Yes | 50  55.6% | 39  43.3% | 1.264 | 1 | 0.261 |
|  | No | 0  0% | 1  1.1% |  |  |  |

## Supplementary Figures

**
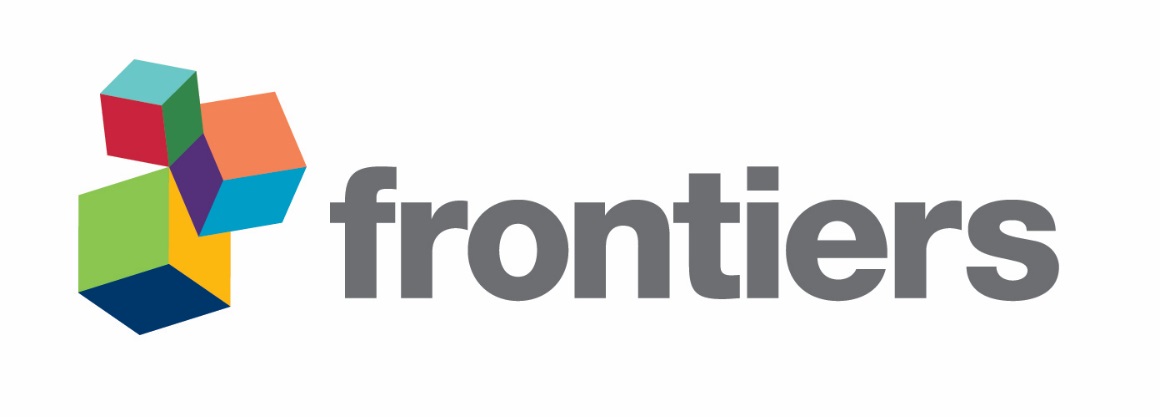
**
